# Review of Serum Biomarkers and Models Derived from Them in HBV-Related Liver Diseases

**DOI:** 10.1155/2020/2471252

**Published:** 2020-07-21

**Authors:** JianPing Wu, WeiLin Mao

**Affiliations:** Department of Clinical Laboratory, The First Affiliated Hospital, College of Medicine, Zhejiang University, Hangzhou, Zhejiang Province 310003, China

## Abstract

A series of predictive scoring systems is available for stratifying the severity of conditions and assessing the prognosis in patients with HBV-related liver diseases. We show nine of the most popular serum biomarkers and their models (i.e., serum cystatin C, homocysteine, C-reactive protein, C-reactive protein to albumin ratio, aspartate aminotransferase to platelet ratio index, fibrosis index based on four factors, gamma-glutamyl transpeptidase to platelet ratio, albumin-bilirubin score, and gamma-glutamyl transpeptidase to albumin ratio) that have gained great interest from clinicians. Compared with traditional scoring systems, these serum biomarkers and their models are easily acquired, simple, and relatively inexpensive. In the present review, we summarize the latest studies focused on these serum biomarkers and their models as diagnostic and prognostic indexes in HBV-related liver diseases.

## 1. Introduction

Hepatitis B virus (HBV) infection is a worldwide epidemic. According to WHO, ~2 billion people worldwide have been infected with HBV, of whom 240 million have chronic HBV infection (CHB) [1]. In total, ~1 million people die each year from HBV-related complications [2]. China has a high prevalence of HBV infection, with ~93 million patients with CHB, and 60% of liver cirrhosis cases and 80% of hepatocellular carcinoma cases are caused by HBV infection [3]. HBV-related acute-on-chronic liver failure (HBV-ACLF), HBV-related liver cirrhosis (HBV-LC), and HBV-related hepatocellular carcinoma (HBV-HCC) represent severe end-stage liver diseases with rapid progression and extremely high mortality [4]. Early identification and precise evaluation of the severity and stages of these diseases will help improve clinical management to decrease the mortality.

Several scoring systems are available to assess liver function and the severity of liver injury, such as the Child-Pugh (CP) score or the model for end-stage liver disease (MELD) score. However, some limitations of these two scores have been described [5, 6]. Recently, some models, indices, or scores derived from serum biomarkers or combinations of these serum biomarkers have been proposed, such as serum cystatin C (CysC), homocysteine (Hcy), C-reactive protein (CRP), aspartate aminotransferase to platelet ratio index (APRI), fibrosis index based on four factors (FIB-4), gamma-glutamyl transpeptidase to platelet ratio (GPR), albumin-bilirubin score (ALBI), gamma-glutamyl transpeptidase to albumin ratio (GAR), and CRP to albumin ratio (CAR), and used in many HBV-related liver diseases, including CHB, HBV-ACLF, HBV-LC, and HBV-HCC, to evaluate their diagnostic and prognostic potential. Compared with traditional scoring systems, these serum biomarkers and their models are easily acquired, simple, and relatively inexpensive. Several studies have reported that these serum biomarkers and their models can complement other scoring systems to monitor and assess the severity and progression of HBV infection. However, the data on this issue are still controversial.

This review summarizes the results of the latest research on these serum biomarkers and their models as diagnostic and prognostic indices in HBV-related liver diseases. Furthermore, we examine the future directions in this field of research.

### 1.1. Serum Cystatin C (CysC)

Serum CysC is a cysteine proteinase inhibitor produced at a constant rate and freely filtered by the glomerular membrane. Several studies suggest that CysC is an accurate indicator of mild renal dysfunction in contrast to classic biomarkers such as the serum creatinine (Cr) or Cr-based formulae [7, 8]. Previous studies have demonstrated that CysC could be a useful predictor of acute kidney injury in patients with cardiac surgery [9], advanced liver diseases [10], and undergoing liver transplantation [11]. Additionally, CysC has been recently found to be a good biomarker of worse outcomes in patients with LC [12, 13].

Wan et al. [14] developed a novel prognostic index combining CysC with total bilirubin (TBil) that was a strong indicator for predicting the 3-month mortality in HBV-ACLF patients. It is noteworthy that in this study, the prognostic index had a higher accuracy than the CP or MELD scores when predicting the mortality of HBV-ACLF patients who had normal Cr levels.

Recently, Wu et al. [15] reported that CysC and the MELD score were positively correlated; higher CysC and MELD scores were also found to be significantly associated with death in all HBV-related decompensated cirrhosis (HBV-DeCi) patients. However, only CysC was an independent factor predicting worse prognosis in patients with normal Cr levels.

### 1.2. Serum Homocysteine (Hcy)

Serum Hcy is a sulfur-containing amino acid that is formed as an intermediary during hepatic methionine metabolism. It may either be remethylated to methionine or catabolized in the transsulfuration pathway to cysteine. Serum Hcy is well-documented as a predictor for cardiovascular diseases [16–18]. The liver plays a central role in Hcy synthesis and metabolism. Thus, it is conceivable that in liver damage situations, alterations in Hcy may occur [19–22].

Zhu and Ma [23] showed that serum levels of Hcy positively correlated with the MELD score and Cr, and there was an inverse correlation estimated for the glomerular filtration rate in the ACLF group. Furthermore, increased Hcy was associated with a worse prognosis in these patients.

### 1.3. Serum C-Reactive Protein (CRP)

Serum CRP is an acute phase protein found in the blood stream, the levels of which increase in response to inflammation. It has also been extensively studied in coronary artery diseases, malignant tumors, tissue necrosis, and bacterial translocation [24–27]. Several studies have been performed on the association of CRP with the severity of inflammation in liver disease, such as fatty liver and chronic hepatitis C (CHC) [28–31]. Few studies have been performed to examine the value of the CRP in assessing the conditions of liver function damage among HBV-related liver diseases.

Zhu et al. [32] showed that CRP and the leukocyte count were positively correlated, but there were no correlations between CRP and the MELD score. The authors suggested that CRP, similar to the MELD score, is an additional independent predictor of short-term mortality. A combination of CRP and the MELD score could increase the prediction efficiency to 92%.

### 1.4. CRP to Albumin Ratio (CAR)

CAR, which is calculated as CRP level divided by the albumin level, was initially used to assess the outcome of patients with acute medical admissions and sepsis [33, 34]. The prognostic ability of CAR has been recently reported in patients with infection, malignancy, and other diseases [35–38]. However, to date, the clinical usefulness of CAR in HBV-related diseases has only been investigated in a limited number of studies.

Huang et al. [39] found that an increased CAR was significantly associated with worse outcomes in HBV-DeCi patients. CAR was first identified as a better prognostic factor compared with the MELD and CP scores using time-dependent ROC curves and time-dependent DeCi.

In a following investigation, Wang et al. [40] showed that the CAR, MELD, and CP scores were positively correlated. The CAR and MELD scores were also found to be a significant predictor of mortality in HBV-DeCi patients. Their analysis suggests that CAR had more accuracy than that of CRP alone in terms of its prognostic ability in HBV-DeCi patients.

### 1.5. Albumin-Bilirubin Score (ALBI)

The ALBI score was initially reported by Johnson et al. to estimate liver function status in HCC patients [41]. The ALBI score involves only 2 objective parameters, albumin and TBil, and it is calculated by the following formula: ALBI score = (log_10_TBil × 0.66) + (albumin×−0.085) [41]. The ALBI score has recently been reported to assess liver dysfunction and prognosis in patients with primary biliary cholangitis and upper gastrointestinal bleeding [42, 43]. Furthermore, the ALBI score has been investigated in several HBV-related liver diseases and has also been proposed as a predictor of worse prognosis in patients with these conditions.

Chen et al. [44] reported that the ALBI score was effective for predicting 1-, 2-, and 3-year mortality in HBV-related LC patients and superior to the CP and MELD scores.

During the same year, Chen and Lin [45] showed that the ALBI score and the MELD and CP scores were positively correlated in ACLF patients. They suggested that both the ALBI and MELD scores were independent predictors of short-term mortality. The combination of the ALBI and MELD scores provided better prediction of mortality than that of the MELD score alone in ACLF patients.

Next, Lei et al. [46] also demonstrated that the ALBI score is a strong indicator of the severity of liver function damage in patients with HBV-ACLF, HBV-LC, or HBV-HCC similar to the CP and MELD scoring systems. A higher ALBI was associated with worse prognosis.

Qi [47] reported that a high ALBI score may be used as an accurate predictor of 1-month mortality in HBV-DeCi patients and may assist physicians in determining treatment options.

One year thereafter, Wang et al. [48] revealed the ALBI score as a predicting index with a good performance to assess long-term prognosis and provide a better prognostic ability compared to MELD scores in patients with HBV-LC. However, the performance of ALBI was not superior to the CP score.

Fujita et al. [49] reported that the ALBI score can be used to stage liver fibrosis in CHB patients and differentiate cirrhotic from noncirrhotic status with an accuracy that is comparable to that of other serum markers. It is noteworthy that higher ALBI scores predicted a worse HCC-free survival.

In a prospective study including 229 HCC patients who underwent hepatectomy, Zou et al. [50] reported that the ALBI score provided better prediction of postoperative outcomes than the CP score. Moreover, the combination of future liver remnants and the ALBI score has the advantage of predicting postoperative morbidity and posthepatectomy liver failure (PHLF) in HBV-HCC patients undergoing hepatic resection compared with future liver remnants or ALBI alone.

More recently, Mai et al. [51] reported that the ALBI-APRI score is an effective and new predictive index of PHLF for HBV-HCC, and its accuracy in predicting the risk of PHLF is better than that of the CP, ALBI, and APRI scores.

### 1.6. Gamma-Glutamyl Transpeptidase to Platelet Ratio (GPR)

Lemoine et al. [52] first proposed GPR as a more accurate routine laboratory biomarker than APRI and FIB-4 for staging liver fibrosis in CHB patients in West Africa in June 2015. The formula for calculating GPR is as follows: GPR = [(GGT/ULN)/platelet count] × 100 (ULN = upper limit of normal) [52]. Several studies have attempted to validate the diagnostic accuracy of GPR for detecting HBV-related fibrosis, but their results were inconsistent.

Li et al. [53] reported that GPR was better than APRI and FIB-4 for assessing hepatic fibrosis in CHB patients with high HBV DNA (≥5 log10 copies/mL) and normal or mildly elevated ALT (≤2 times ULN) in a Chinese population. It is recommended to make GPR widely available to this particular population.

Next, Ren et al. [54] demonstrated that GPR has advantages compared with APRI in identifying fibrosis, significant fibrosis, and extensive fibrosis, and it was comparable with FIB-4 in diagnostic performance.

Moreover, Liu et al. [55] showed that GPR, APRI, and FIB-4 all have moderate accuracy in the prediction of different levels of liver fibrosis, though GPR has the best performance regardless of the HBeAg state.

In agreement with these findings, Lee et al. [56] also showed that GPR and FIB-4 can significantly screen the liver fibrosis status in CHB patients and that GPR displayed the best diagnostic performance.

A meta-analysis by Lian et al. [57] reported that GPR has a moderate diagnostic accuracy for predicting HBV-related significant fibrosis, severe fibrosis, and cirrhosis. Although the diagnostic accuracy of GPR is not high, GPR should be widely used in clinical practice for its strengths.

Wang et al. [58] demonstrated that GPR has a significantly higher diagnostic accuracy compared with APRI and FIB-4 in Chinese patients with CHB. Age and elevated AST and TBil may affect the diagnostic accuracy of GPR.

Next, Zhang et al. [59] indicated that the accuracy of GPR to diagnose significant fibrosis and extensive fibrosis was comparable to that of APRI, while its accuracy to diagnose cirrhosis was better. Additionally, the responsiveness of GPR was greater than that of APRI and FIB-4 during treatment with nucleoside/nucleotide analogues.

Nevertheless, Li et al. [60], Huang et al. [61], and Lu et al. [62] indicated that although GPR is a novel diagnostic model for liver fibrosis or cirrhosis, it does not display advantages compared to APRI and FIB-4 for identifying significant fibrosis, severe fibrosis, and cirrhosis in Chinese CHB patients.

Next, Hu et al. [63] demonstrated that GPR is more accurate, sensitive, and easy to use than FIB-4 and APRI, and it can significantly improve the sensitivity and specificity of hepatic fibrosis diagnosis in CHB when combined with FIB-4 or APRI.

During the same year, Desalegn et al. [64] found that APRI, FIB-4, and GPR had good diagnostic performance for assessing liver fibrosis or cirrhosis in CHB patients in East Africa. However, the sensitivities of all the tests were poor.

Next, Hamidi et al. [65] showed that APRI, FIB-4, and GGT were successful for the detection of liver fibrosis in CHB patients. GPR and FIB-4 may be useful for predicting advanced fibrosis in cases with CHB.

Wang et al. [66] demonstrated that age may influence the diagnostic thresholds and performance of APRI, FIB-4, and GPR for significant fibrosis in CHB patients. These scores performed poorly for identifying significant fibrosis in younger patients (≤30 years).

Wang et al. [67] found that a GPR score ≥ 0.84 represents a risk factor for the adverse prognosis of HBV-HCC after hepatic resection, and GPR served as a strong predictive factor for HBV-HCC overall survival.

In a following investigation, Pang et al. [68] also demonstrated that a low GPR may correlate with good survival and recurrence-free survival after therapy in HBV-HCC patients. Moreover, the GPR can be applied to evaluate liver function and stage of liver fibrosis and to predict the prognosis in these patients.

Park et al. [69] showed that GPR could serve as an effective index to assess the risk of HCC development in CHB patients.

Liu et al. [70] demonstrated that age, GPR, and the MELD score are independent risk factors associated with HBV-ACLF prognosis. Incorporating GPR into the MELD score may improve the prognostic assessment of patients with HBV-ACLF.

Finally, Wang et al. [71] and Yu et al. [72] proposed that GPR may be used as an accurate and convenient alternative to liver biopsy in evaluating liver fibrosis and inflammation in CHB patients.

### 1.7. Aspartate Aminotransferase to Platelet Ratio Index (APRI)

APRI was suggested by Wai et al. [73] to predict fibrosis and cirrhosis in CHC. The formula for the calculation of APRI is as follows: APRI = [(AST/ULN)/platelet count] × 100 [73]. In 2015, WHO published its first guideline on the management of CHB. They recommend the use of APRI as a noninvasive tool to detect liver cirrhosis in resource-limited settings [74]. Several studies have assessed the role of APRI in CHB patients, though the results remain controversial.

Wai et al. [75] reported that the accuracy of the best predictive models for APRI was only modest for significant fibrosis and cirrhosis in CHB patients.

Shin et al. [76] found that APRI might be a readily available index for predicting significant fibrosis, with CHB and APRI potentially being used to decrease the number of liver biopsies.

During the same year, Lin et al. [77] indicated that APRI is a good estimator of liver fibrosis and is more accurate for CHC than CHB.

Furthermore, Zhang et al. [78] suggested that an APRI ≥ 1.5 in combination with a hyaluronic acid cut − off point > 300 ng/mL can detect moderate to severe fibrosis in CHB patients.

In a subsequent investigation, Hung et al. [79] demonstrated that APRI could serve as a surrogate for the evaluation of liver functional reserve, assessment of hepatic fibrosis, and prediction of survival for HBV-related HCC patients.

Next, Liu et al. [80] proposed that patient age may influence cirrhosis, and they claimed that APRI may have better accuracy for CHB patients, especially patients > 5 years.

Additionally, Lesmana et al. [81] showed that transient elastography (TE) and APRI both have good diagnostic accuracy for detecting F3 and greater fibrosis. APRI is better than TE for detecting F2 and greater fibrosis. A combination of both methods does not significantly increase the diagnostic accuracy and is not recommended.

Wang et al. [82] indicated that both FIB-4 and APRI are useful in identifying those without significant fibrosis with >85% accuracy. Stratifying those with FIB − 4 ≤ 1.45 or APRI ≤ 0.5 would have reduced the number of biopsies performed in the entire cohort by 72%.

Gumusay et al. [83] also suggested that hyaluronic acid, the enhanced liver fibrosis panel, and APRI have better diagnostic values for predicting moderate to severe fibrosis. However, a liver biopsy was needed for the detection of mild fibrosis in CHB patients.

During the same year, Ucar et al. [84] reported that APRI, FIB-4, and Forn's index have better diagnostic value in patients with significant fibrosis than the diagnostic value in those with no/minimal fibrosis.

Shrivastava et al. [85] concluded that APRI and FIB-4 can be utilized in combination as a screening tool to monitor CHB patients, especially in resource-limited settings.

Xiao et al. [86] showed that APRI and FIB-4 correlate with HBV-related fibrosis levels in HCC patients. However, the accuracy of APRI and FIB-4 for predicting HBV-related LC in adult HCC patients is not very high.

In contrast, Houot et al. [87] indicated that APRI had a lower performance than FIB-4, TE, and the FibroTest in CHC and CHB.

Moreover, Huang et al. [88] also found that APRI and FIB-4 had a worse predictive ability for identifying significant, advanced LF and cirrhosis than real-time ultrasound elastography in CHB patients in China, with lower sensitivity and accuracy.

Jin et al. [89] performed a meta-analysis to determine the accuracy of APRI in the prediction of hepatic fibrosis. The summarized results suggested that APRI displayed limited value in determining HBV-related fibrosis and cirrhosis.

A systemic review and meta-analysis were performed on data from 39 studies including 16 articles focused on APRI alone, 21 articles on APRI and FIB-4, and two articles on FIB-4 for detecting different levels of liver fibrosis in patients with CHB. Xiao et al. [90] demonstrated that APRI and FIB-4 have moderate diagnostic accuracy for predicting fibrosis. Although the diagnostic accuracy of FIB-4 and APRI is not high, they can still be considered as a choice for the prediction of fibrosis caused by CHB infection in regions with limited healthcare resources.

Finally, Mao et al. [91] showed that elevated APRI was associated with an increased severity of liver disease and short-term mortality in HBV-LC patients. Their results indicated that APRI and the MELD score were two independent models for predicting adverse outcomes. A combination of the MELD score and APRI further augmented the predicting power.

### 1.8. Fibrosis Index Based on Four Factors (FIB-4)

The FIB-4 index was initially developed by Sterling et al. to predict significant fibrosis in patients with HCV/HIV coinfection [92]. The formula for the calculation of FIB-4 is as follows: FIB − 4 = age × AST/platelet count × [ALT]^1/2^ [92]. The FIB-4 index has also been validated in HBV-infected patients in numerous studies, but the diagnostic value of FIB-4 remains controversial.

Mallet et al. [93] stated that the FIB-4 index was accurate in the evaluation of nil-to-moderate fibrosis in CHB.

In a subsequent investigation, Kim et al. [94] pointed out that FIB-4 cut-offs of 1.6 and 3.6 provided a 93.2% negative predictive value and a 90.8% positive predictive value for the detection of cirrhosis, respectively. Based on these results, liver biopsies could be avoided in 70.5% of the study population.

Additionally, Erdogan et al. [95] suggested that the FIB-4 index may be useful in estimating the extent of fibrosis in CHB patients.

Ma et al. [96] demonstrated that APRI, FIB-4, and Lok's model are very effective in staging fibrosis in CHB patients. FIB-4 and Lok's model are the best models to distinguish between extensive and significant fibrosis.

Koksal et al. [97] showed that FIB-4, RPR, and platelet count were better for displaying advanced fibrosis. Moreover, these markers can be used to monitor the response to treatment.

Kim et al. [98] confirmed that not only APRI but also FIB-4 was an effective predictor of HCC development and CHB patient prognosis.

More recently, Lee et al. [99] denoted that FIB-4 is useful for the noninvasive prediction of HCC development, while APRI and GPR were less useful.

Li et al. [100] reported that FIB-4 and APRI predicted the hepatic fibrosis stage with a high degree of accuracy in both CHB and CHC patients. FIB-4 was a slightly better predictor in CHC populations but not in CHB populations.

However, Zhu et al. [101] showed that APRI and FIB-4 were not superior to FibroScan for the diagnosis of significant liver fibrosis and cirrhosis in Western Chinese CHB patients.

Furthermore, Kim et al. [102] indicated that APRI and FIB-4 models were not suitable for use in clinical practice in CHB patients for the assessment of liver fibrosis, especially in gauging improvements in liver fibrosis following therapy.

A meta-analysis by Li et al. [103] indicated that FIB-4 is valuable for detecting significant fibrosis and cirrhosis but has suboptimal accuracy when excluding fibrosis and cirrhosis in HBV-infected patients.

Another meta-analysis by Yin et al. [104] showed that FIB-4 has a relatively high diagnostic value for detecting liver fibrosis in CHB when the diagnostic threshold value was greater than 2.0.

### 1.9. Gamma-Glutamyl Transpeptidase to Albumin Ratio (GAR)

GAR is calculated as GGT level divided by albumin level. It is an accurate and low-priced index to identify patients with obvious fibrosis or cirrhosis.

Li et al. [105] reported that GAR is a simple noninvasive biomarker compared to APRI and FIB-4 to stage significant fibrosis and cirrhosis in CHB patients and represents a novel noninvasive alternative to liver biopsy.

## 2. Conclusions

This review assessed the value of serum biomarkers and their models in HBV-related liver diseases. The significance of serum CysC, serum Hcy, serum CRP, CAR, ALBI score, FIB-4 index, APRI, GPR, and GAR in the diagnosis and prognosis of HBV-related liver diseases has been indicated in many studies, as shown above. The most crucial studies regarding these serum biomarkers and their models are summarized in Table 1.

The last decade has seen significant progress in the development of noninvasive liver disease assessments in CHB patients. These assessments are not only used to identify patients with a high risk of adverse outcomes but also to monitor disease progression and therapeutic responses after interventions. Additionally, several studies also pointed out that these models should be used in combination with other noninvasive indices to achieve more efficient diagnostic and prognostic efficiency. Of course, the clinical utility of these serum biomarkers and their models as diagnostic and prognostic indices in HBV-related liver diseases requires further verification, and concrete steps should be undertaken to define standards that can serve as guidelines for effectively using serum biomarkers and their models as tools for diagnostic and prognostic assessments.

## Figures and Tables

**Table 1:** Summary of studies investigating the diagnostic and prognostic roles of serum biomarkers and their models in patients with HBV-related liver diseases.

Study type	Study	Sample size	Main findings	Refs.
Serum CysC				
Prospective	Wan et al. (2015)	72 ACLF	CysC plus total bilirubin can predict the short-term outcomes in HBV-ACLF patients.	[14]
Retrospective	Wu et al. (2019)	75 DeCi	High CysC can be considered a simple marker of 3-month mortality in HBV-DeCi patients.	[15]
Serum Hcy				
Retrospective	Zhu et al. (2018)	52 ACLF, 52 CHB, and 65 HCs	Serum Hcy can be an effective predictor of a worse prognosis in HBV-ACLF patients.	[23]
Serum CRP				
Retrospective	Zhu et al. (2017)	140 DeCi	Elevated CRP was associated with short-term mortality in HBV-DeCi patients.	[32]
CAR				
Retrospective	Huang et al. (2017)	329 DeCi	CAR was associated with the prognosis of HBV-DeCi and is superior to the MELD and CP scores in HBV-DeCi mortality prediction.	[39]
Retrospective	Wang et al. (2019)	113 DeCi	High CAR was associated with 1-month mortality in HBV-DeCi patients.	[40]
ALBI score				
Retrospective	Chen et al. (2017)	806 LC	ALBI is a simple predictor of long-term mortality in LC patients compared with the CP and MELD scores.	[44]
Retrospective	Chen et al. (2017)	84 AoCLF, 56 CHB, and 48 HCs	A high ALBI score may be used as a predictor for the 3-month mortality in HBV-ACLF patients.	[45]
Retrospective	Lei et al. (2018)	138 ACLF, 130 LC, and 127 HCC	ALBI showed parallel tendencies to the CP and MELD scores in HBV-ACLF, HBV-LC, and HBV-HCC patients.	[46]
Retrospective	Qi et al. (2018)	81 DeCi	ALBI is an accurate index to predict 1-month outcomes in HBV-DeCi patients.	[47]
Retrospective	Wang et al. (2019)	398 LC	The ALBI score accurately predicts the severity and prognosis of HBV-LC patients, and its prognostic performance was superior to the MELD score.	[48]
Retrospective	Fujita et al. (2019)	91 CHB	The ALBI score can be used for liver fibrosis staging in CHB, and a lower ALBI score predicts better HCC-free survival.	[49]
Prospective	Zou et al. (2018)	229 HCC	The ALBI score was a superior predictive value of postoperative outcomes over the CP score.	[50]
Retrospective	Mai et al. (2019)	1,055 HCC	The ALBI-APRI score is an accurate predictive model of posthepatectomy liver failure for HCC patients.	[51]
GPR				
Retrospective	Lemoine et al. (2016)	135 treatment-naïve CHB	GPR is a more accurate index than that of APRI and FIB-4 to stage liver fibrosis in CHB patients.	[52]
Retrospective	Li et al. (2016)	1521 CHB	GPR shows advantages in assessing hepatic fibrosis in patients with HBV DNA ≥ 5 log10 copies/mL and ALT ≤ 2 times ULN compared with APRI and FIB-4.	[53]
Retrospective	Ren et al. (2017)	160 treatment-naïve CHB	GPR is a simple laboratory marker to stage liver fibrosis in CHB patients.	[54]
Retrospective	Liu et al. (2018)	2016 CHB	GPR had the best performance in predicting different stages of fibrosis compared with APRI and FIB-4.	[55]
Retrospective	Lee et al. (2018)	278 CHB	FIB-4 and GPR are a simple model for evaluating the severity of liver fibrosis in CHB patients.	[56]
Meta-analysis	Lian et al. (2019)	10 studies	GPR has moderate diagnostic accuracy for predicting HBV-related significant fibrosis, severe fibrosis, and cirrhosis.	[57]
Prospective	Wang et al. (2016)	312 CHB	GPR was a more reliable laboratory marker than APRI and FIB-4 for predicting the stage of liver fibrosis in Chinese CHB patients.	[58]
Retrospective	Zhang et al. (2018)	1168 CHB	GPR is considered a simple index for the diagnosis of liver fibrosis and the dynamic assessment of treatment responses in Chinese CHB patients.	[59]
Retrospective	Li et al. (2016)	372 CHB	GPR does not display advantages compared to APRI and FIB-4 for identifying significant fibrosis, severe fibrosis, and cirrhosis in CHB patients.	[60]
Retrospective	Huang et al. (2017)	256 CHB	GPR does not show advantages compared to APRI and FIB-4 in assessing liver fibrosis, advanced fibrosis, or cirrhosis in Chinese CHB patients.	[61]
Retrospective	Lu et al. (2018)	397 treatment-naïve CHB	GPR does not appear to represent a significant step forward compared with FIB-4 and APRI.	[62]
Retrospective	Hu et al. (2017)	390 treatment-naïve CHB	GPR can improve the sensitivity and specificity of hepatic fibrosis diagnosis in CHB when combined with FIB-4 or APRI.	[63]
Retrospective	Desalegn et al. (2017)	582 treatment-naïve CHB	APRI, FIB-4, and GPR had good diagnostic properties in CHB patients, though the sensitivities of the tests were low.	[64]
Retrospective	Hamidi et al. (2019)	202 CHB	GPR and FIB-4 may be useful for predicting advanced fibrosis in CHB.	[65]
Retrospective	Wang et al. (2019)	496 CHB	Age may influence the diagnostic thresholds and performance of APRI, FIB-4, and GPR for significant fibrosis in CHB patients.	[66]
Retrospective	Wang et al. (2016)	357 HCC	GPR served as an independent predictive factor for HBV-HCC overall survival.	[67]
Retrospective	Pang et al. (2016)	182 HCC	GPR is a simple predictor of outcomes in HBV-HCC.	[68]
Retrospective	Park et al. (2017)	1109 CHB	GPR can serve as a noninvasive index to assess the risk of HCC development in CHB patients.	[69]
Retrospective+prospective	Liu et al. (2018)	355 ACLF	Incorporating GPR into MELD may provide more accurate survival prediction in HBV-ACLF patients.	[70]
Retrospective	Wang et al. (2018)	519 CHB	GPR can be an effective model for predicting liver inflammation in CHB.	[71]
Retrospective	Yu et al. (2019)	160 treatment-naïve CHB	GPR is an effective model to assess liver fibrosis and inflammation activity.	[72]
APRI				
Retrospective	Wai et al. (2006)	218 treatment-naïve CHB	APRI was not able to accurately predict cirrhosis in CHB patients.	[75]
Retrospective	Shin et al. (2008)	264 CHB	APRI may be an effective index for predicting significant fibrosis in CHB.	[76]
Retrospective	Lin et al. (2008)	48 CHB, 40 CHC, and 9 HCs.	APRI could be used to decrease the number of liver biopsies.	[77]
Retrospective	Zhang et al. (2008)	137 CHB	APRI combined with hyaluronic acid could achieve a better diagnostic accuracy of liver fibrosis.	[78]
Retrospective	Hung et al. (2010)	76 HCC	APRI can be an effective model for assessing liver fibrosis and predicting survival in HBV-HCC patients.	[79]
Cross-sectional study	Liu et al. (2011)	623 CHB	APRI may have better accuracy for CHB patients, especially patients > 35 years in age.	[80]
Cross-sectional study	Lesmana et al. (2011)	117 CHB	APRI is a simple index to screen liver fibrosis in the primary care setting.	[81]
Retrospective	Wang et al. (2013)	239 CHB	Both FIB-4 and APRI are useful for the identification of those without significant fibrosis. However, they have a poor positive predictive value.	[82]
Prospective	Gumusay et al. (2013)	58 CHB and 30 HCs	Combination of the enhanced liver fibrosis panel and APRI has a better diagnostic value in predicting fibrosis ≥ F3 in CHB patients.	[83]
Retrospective	Ucar et al. (2013)	73 CHB	APRI, FIB-4, and Forn's index have a better diagnostic value in patients with significant fibrosis than the diagnostic value in those with no/minimal fibrosis.	[84]
Cross-sectional study	Shrivastava et al. (2013)	52 CHB and 25HCs	APRI and FIB-4 can be utilized in combination as screening tools to monitor CHB patients.	[85]
Retrospective+prospective	Xiao et al. (2016)	2176 LC accompanied with HCC	APRI and FIB-4 correlate with liver fibrosis, but they have low accuracy for predicting HBV-LC in HCC patients.	[86]
Systematic review+meta-analysis	Houot et al. (2016)	71 studies	APRI had lower performance than that of FIB-4, TE, and FibroTest in CHC and CHB.	[87]
Retrospective	Huang et al. (2019)	91 CHB	Real-time ultrasound elastography is reliable for the assessment of liver fibrosis in CHB patients and has better discrimination power than that of APRI and FIB-4.	[88]
Meta-analysis	Jin et al. (2012)	9 studies	APRI displayed limited value in identifying HBV-related fibrosis and cirrhosis.	[89]
Systematic review+meta-analysis	Xiao et al. (2015)	39 articles	APRI and FIB-4 can identify HBV-related fibrosis with a moderate sensitivity and accuracy.	[90]
Retrospective	Mao et al. (2016)	193 CHB and 88 HCs	APRI can be a simple predictor of adverse outcomes in HBV-DeCi patients.	[91]
FIB-4				
Retrospective	Mallet et al. (2009)	138 CHB	FIB-4 is a cheap index to screen liver fibrosis in CHB.	[93]
Cross-sectional study	Kim et al. (2010)	668 CHB	FIB-4 may reduce the need for liver biopsy in the majority of CHB patients.	[94]
Retrospective	Erdogan et al. (2013)	221 CHB	FIB-4 may be useful in estimating the extent of fibrosis in CHB patients.	[95]
Retrospective	Ma et al. (2013)	1168 CHB	FIB-4 and Lok's model are the most effective models for distinguishing significant and extensive fibrosis.	[96]
Retrospective	Koksal et al. (2016)	228 CHB	FIB-4, RPR, and platelet count were better for demonstrating advanced fibrosis.	[97]
Retrospective	Kim et al. (2016)	542 CHB	APRI and FIB-4 were effective predictors of HCC development and CHB patient prognosis.	[98]
Retrospective	Kim et al. (2019)	444 CHB	FIB-4 is useful for the noninvasive prediction of HCC development, while APRI and GPR were less useful.	[99]
Retrospective	Li et al. (2014)	284 CHB	FIB-4 and APRI predicted the liver fibrosis stage with a high degree of accuracy in both CHB and CHC patients.	[100]
Prospectively	Zhu et al. (2011)	175 CHB	APRI and FIB-4 were not superior to FibroScan for the diagnosis of significant liver fibrosis and cirrhosis in Western Chinese CHB patients.	[101]
Retrospective	Kim et al. (2016)	575 CHB	FIB-4 is not reliable for detecting the regression of fibrosis following antiviral treatment.	[102]
Meta-analysis	Li et al. (2014)	22 studies	FIB-4 is valuable for detecting significant fibrosis and cirrhosis in HBV-infected patients but has suboptimal accuracy when excluding fibrosis and cirrhosis.	[103]
Meta-analysis	Yin et al. (2017)	26 studies	FIB-4 has a high diagnostic value for detecting liver fibrosis in CHB patients when the diagnostic threshold value is greater than 2.0.	[104]
GAR				
Retrospective	Li et al. (2017)	822 CHB	GAR shows obvious advantages when predicting significant fibrosis and cirrhosis in Chinese CHB patients compared with APRI and FIB-4.	[105]

Abbreviations: ACLF—acute-on-chronic liver failure; CHB—chronic hepatitis B; CHC—chronic hepatitis C; CP—Child-Pugh score; DeCi—decompensated cirrhosis; HBV—hepatitis B virus; HCC—hepatocellular carcinoma; HCs—healthy controls; LC—liver cirrhosis; MELD—model for end-stage liver disease; APRI—aspartate aminotransferase to platelet ratio index; FIB-4—fibrosis index based on the four factors; GPR—gamma-glutamyl transpeptidase to platelet ratio; TE—transient elastography; ULN—upper limit of normal.

## Data Availability

This is a review article and it does not need data.

## Abbreviations

ACLF: Acute-on-chronic liver failure
APRI: Aspartate aminotransferase to platelet ratio index
CHB: Chronic hepatitis B
CHC: Chronic hepatitis C
CP: Child-Pugh score
Cr: Creatinine
DeCi: Decompensated cirrhosis
FIB-4: Fibrosis index based on four factors
GPR: Gamma-glutamyl transpeptidase to platelet ratio
HBV: Hepatitis B virus
HCC: Hepatocellular carcinoma
HCs: Healthy controls
LC: Liver cirrhosis
MELD: Model for end-stage liver disease
TE: Transient elastography
TBil: Total bilirubin
ULN: Upper limit of normal.
